# Regaining independence: a grounded theory study of the process of rehabilitation changes in patients with hemiplegic stroke

**DOI:** 10.1080/17482631.2025.2581404

**Published:** 2025-11-09

**Authors:** Huaqing Liu, Zhe Li, Shiyou Fu, Zhengjia Ren

**Affiliations:** a Department of Rehabilitation Medicine, The Third Affiliated Hospital of Chongqing Medical University, Chongqing, People's Republic of China; b Department of Clinical Psychology, The Third Affiliated Hospital of Chongqing Medical University, Chongqing, People's Republic of China

**Keywords:** Poststroke, rehabilitation, independence, multidimensional system, grounded theory

## Abstract

**Background:**

Research focusing on the rehabilitation experiences of stroke patients in China remains limited. Understanding their recovery journey is essential for improving care strategies and enhancing patients’ well-being. This study aimed to explore the psychosocial mechanisms and processes underlying the rehabilitation of hemiplegic stroke patients.

**Methods:**

This study employed a constructivist grounded theory approach to understand the rehabilitation experiences of 13 hemiplegic stroke patients, recruited via theoretical sampling for in-depth interviews. Data were analyzed concurrently using the constant comparison method until theoretical saturation was reached, culminating in the construction of a theoretical model explaining their process of change.

**Results:**

During the transition from illness to rehabilitation, hemiplegic stroke patients struggled with the lack of independence. With the support of a multidimensional system, these patients can partially regain independence**.**

**Conclusion:**

This study revealed that the development of a holistic supportive services model can help patients receive timely and effective positive support. In future rehabilitation services, the multidimensional service model needs to be considered, and various facilitating factors should be improved to provide comprehensive and systematic rehabilitation services to patients.

## Introduction

Stroke, also known as an acute cerebrovascular event, is a localized or comprehensive brain dysfunction syndrome caused by an acute cerebrovascular circulation disorder and is characterized by high morbidity, a high disability rate and a high recurrence rate (Yanan et al., 2021). Stroke has become the primary cause of global dysfunction and disability and is usually accompanied by extensive impairment of physical, cognitive, social, and emotional functions (Kuriakose & Xiao, 2020).

An increasing number of researchers are calling for timely rehabilitation for stroke patients; however, rehabilitation currently focuses more on the recovery of physical function. A growing body of evidence confirms that psychosocial rehabilitation plays an equally important role in the rehabilitation of stroke patients (Geler Külcü et al., 2022). Previous studies have shown that comprehensive psychosocial rehabilitation can significantly improve patient recovery and quality of life (Gurková et al., 2023; Yoon et al., 2021). Approximately 70–80% of stroke patients in China cannot live independently due to disability (Chinese Society of Neurology et al., 2017); thus, they are physically and emotionally dependent on their caregivers, which has an enormous impact on their lives. One systematic review revealed that stroke can cause serious disruptions that may lead to physical dysfunction, uncertainty, reduced independence, loss of control, and frustration (Sarre et al., 2014). A systematic review of qualitative studies revealed that stroke survivors experienced distrust of their bodies and discontinuity of the self before and after stroke and continued to struggle with uncertainty and discontinuity after discharge. These studies revealed the consistent experience of stroke patients, that is, the loss of body independence after stroke, and that patients hope to restore their independence through various efforts (Lou et al., 2017).

One systematic review by Woodman et al. focused on the personal experiences of stroke survivors, revealing that the disease and its complications can severely destroy the independence, social lives and interpersonal relationships of survivors and that stroke can cause related problems such as fatigue, cognitive decline, functional disability, difficulty walking, decreased concentration, transportation difficulties, and economic constraints, which also affect and limit the independence of stroke patients (Woodman et al., 2014). The studies included in this review revealed that the difficulty due to limited independence caused by the illness and the lack of effective support for disability‐related problems increase the likelihood of stroke patients failing to regain independence after stroke, which undoubtedly limits their ability to achieve comprehensive and effective rehabilitation (Törnbom et al., 2018). Several recent studies have shown that stroke survivors can build confidence through various forms of psychosocial support, which can help them gain more independence and a sense of control (Greenwood et al., 2010; Villain et al., 2017). It is very important to help stroke patients gradually regain their independence during the rehabilitation period, as this can enhance patients’ subjective feelings and reduce the burden of care on caregivers (Karahan et al., 2014).

At present, few studies have focused on the experience of stroke patients during the rehabilitation process in China. Understanding their experiences is critical and can help us better understand their needs and how caregivers and medical workers can better provide holistic care, which can improve patients’ subjective experiences. Qualitative research can provide a better understanding of the transformation process in stroke patients. This change process can provide more information for evidence‐based practice and provide rehabilitation professionals with the opportunity to develop a more in‐depth understanding of patients and to reflect on how their clinical practice can more effectively strengthen clinical rehabilitation strategies to improve patients’ rehabilitation outcomes. Therefore, the present study aimed to use grounded theory to explore the psychosocial processes involved in the rehabilitation of patients with hemiplegic stroke and to develop a theoretical model explaining their journey of struggling to regaining independence within the Chinese context.

## Methods

### Study design

This study employed a constructivist grounded theory approach, as articulated by Charmaz (Charmaz, 2000). This methodology is rooted in a subjective epistemology, which assumes that the researcher and participants co‐create knowledge and acknowledges that reality is a social construction (Ghezeljeh & Emami, 2009).

Therefore, this approach is ideal for exploring the subjective journey of rehabilitation from the participants' perspectives. Through in‐depth interviews, our goal was not simply to describe patient experiences but to uncover the underlying social and psychological processes of their recovery. By allowing emerging data to guide the analysis, we developed a substantive theory that explains how patients navigate the complex transition from a loss of independence toward regaining it.

### Participants

Participants were recruited from the Department of Rehabilitation Medicine at a university‐affiliated hospital.

#### **Inclusion and exclusion criteria**

To be included in the study, participants had to meet the following criteria: (1) a diagnosis of a first‐time hemiplegic stroke; (2) admission to the rehabilitation department for recovery; (3) medical stability; and (4) the ability to communicate clearly and provide informed consent. Patients were excluded if they had: (1) severe aphasia or cognitive impairments that would prevent an in‐depth interview; or (2) a pre‐existing diagnosis of a major psychiatric disorder.

#### Participant recruitment

This study utilized a theoretical sampling strategy. Recruitment of eligible patients continued until theoretical saturation was reached, which occurred after a total of 13 participants had been interviewed. No additional participants were recruited once saturation was achieved. The demographic characteristics of the participants are shown in Table I.

**Table I.** Basic demographic information of the participants (*n* = 13).

Variable	Characteristic	*n*
Sex	Male	8
	Female	5
Average age	63.7 (41−80)	
Marital status	Married	11
	Divorced	2
Education level	Bachelor's degree	1
	Junior College	4
	High school	2
	Junior high school	2
	Primary school	4
Occupation	Full‐time work	4
	Retirement	8
	Individual	1
Average monthly income	10,000 yuan or more	1
	5000−8000 yuan	6
	Less than 5,000 yuan	5
	No stable income	1
Residential environment	City	7
	Town	4
	Rural area	2
Damaged location	Cerebral hemispheric area	3
	Basal ganglia region	9
	Fronto‐parietal‐temporal insular region	1
Average Barthel Index	59.23 (10−85)	

### Sampling

In this study, sample size was not predetermined but was instead guided by theoretical sampling. This iterative process involved conducting data collection and analysis concurrently to inform the selection of subsequent participants.

We began by recruiting several patients who met the general inclusion criteria. As these initial interviews were analyzed, emerging concepts‐such as the importance of peer support and the role of family expectations‐guided the recruitment of new participants who could best elaborate on these themes. For instance, to further develop the “peer support” category, we intentionally sought a patient observed to be more socially isolated to understand potential barriers. This process allowed us to purposefully select participants who could help refine and saturate the developing theoretical categories. Sampling continued until theoretical saturation was achieved, the point at which no new significant properties of the core categories were emerging from the data.

### Trustworthiness

To ensure the trustworthiness of this constructivist grounded theory study, we followed Lincoln and Guba’s (1985) framework, including credibility, transferability, dependability, and confirmability (Lincoln et al., 1985). Credibility was enhanced through prolonged engagement, member checking, and data triangulation to ensure authentic representation of participants’ rehabilitation experiences.

Transferability was supported by providing thick descriptions of participants and settings. Dependability was maintained through a detailed audit trail, and confirmability was strengthened via reflexive journaling. These strategies collectively ensured methodological rigor and transparency in developing a grounded theory of the rehabilitation experiences of stroke patients with hemiplegia.

### Data collection and analysis

All interviews were conducted in a quiet room within the hospital by a two‐person team. One interviewer held a doctoral degree in medicine while the other was the head nurse of the Department of Rehabilitation. The interviews with stroke patients lasted between 20 and 45 minutes, with an average duration of 32 minutes. They were initially guided by open‐ended questions, inviting participants to share their feelings, needs, difficulties, and successes following their stroke. During the interviews, the interviewers also posed follow‐up questions based on participants’ responses‐for example, “What are your current difficulties?” Participants were encouraged to speak freely and elaborate on their experiences.

Two researchers independently analyzed the interview data. One holds a medical doctorate with extensive experience in qualitative research methods and has been invited multiple times to share this expertise at various universities. All interview recordings were transcribed into Word documents and then imported into Excel for coding. The researchers employed a constant comparative method to identify connections between conceptual categories, ultimately leading to theory development.

The transcribed data underwent initial coding line by line. Initial coding involves extracting concepts and categories from the data, identifying similarities and differences, and classifying them accordingly. Based on the initial interview content and emerging codes, new participants were recruited to collect additional data for further coding. Concepts and categories were continuously extracted from these data until the relationships between them were refined, theories emerged, and a theoretical explanation of the research problem was established. Throughout the process, the researchers sought feedback from participants to ensure that the resulting theory remained firmly grounded in the data.

## Results

**Figure 1.**
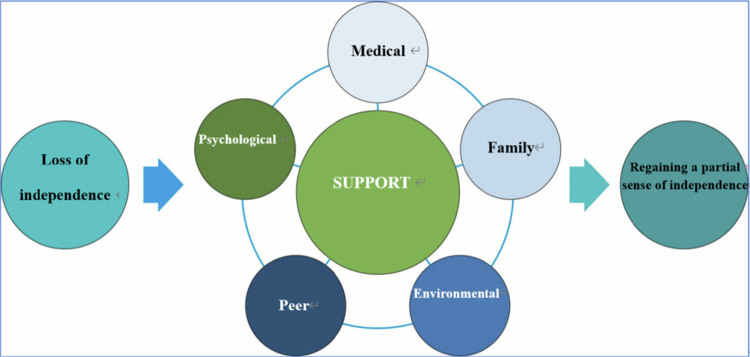
Holistic rehabilitation process model.

Through an iterative process of initial and focused coding, the central process of “Regaining Independence” was constructed from the data. This process describes the journey of hemiplegic stroke patients from an initial state of “Losing Independence” toward “Achieving Partial Independence.” We found this transition was mediated by a core category we termed the “Multidimensional Support System,” which includes medical, family, psychological, peer, and environmental support. This theory was developed by constantly comparing data and refining categories until theoretical saturation was reached. The holistic rehabilitation process model (environmental‐medical‐familial‐social‐psychological‐peer support services model) is presented in Figure 1.

### Loss of independence

Many participants described their personal experiences after stroke. Independence refers to making independent decisions that align with personal values and goals instead of coercion by external forces, which implies having the freedom to govern oneself and exercise self‐determination. Due to the impairment of cognitive and physical functions, stroke patients experienced a sense of loss of control over their own bodies and over their lives. In many cases, they were not only unable to have the freedom to govern themselves but also had to rely on others, which seriously decreased their quality of life, described by two patients as follows:

*“I can’t turn my feet or bend my hands…I feel very anxious now.” (P1)*

*“I can’t accept it, that is, when these things happen to me; I still need the help of others, and I can’t figure out easy things by myself… a*fter my stroke, I even needed help shaving, which was totally unacceptable*... I feel that I am being taken care of by them. No, it should not be a continuous process. In addition, my family takes care of me every day... I feel very bad”. (P2)*

After this patient suffered his stroke, he lost his independence and his sense of control, and he was unable to effectively cope with his new daily life, which reflected his sense of loss of control and frustration in his life. Another patient also complained about a similar feeling of loss of independence:

*“I’m annoyed when I can’t take care of myself. If you can’t do simple things like that, it causes a lot of harm… Like now, I can’t do things such as dressing and eating. None of these can be done….” (P8)*

Almost all patients expressed similar feelings. In the past, they had total control of their lives, but they lost control over their bodies and lives after stroke, which led to worry, anxiety and frustration due to the loss of independence. These factors led the patients to develop a negative view and evaluation of themselves.

### Support from a multidimensional system

Many patients expressed the importance of support for patient recovery. However, the support needed for rehabilitation patients comprises multiple types, including rehabilitation, psychosocial, environmental, and peer support.

#### **Medical support**

The effects of medical rehabilitation helped the patients gain confidence and hope in the process of rehabilitation:

*“(In the first two months), I couldn’t even move, and I couldn’t sit stably. Now I can walk and sit stably...well, I am very confident and hopeful..." (P6)*

The patient mentioned that the support of medical personnel played a vital role in their recovery process.

*“At first (in the beginning), I couldn’t move, but when I could move a little bit later, I was very happy. If my foot moved, I was very happy… According to the guidance of the rehabilitation therapist, they asked me to work harder and encouraged me and gave me hope. Human beings like to be praised; you just want to do well and be praised”. (P8)*

#### **Support from family members**

Another patient also mentioned the importance of family support in his recovery:

*“My family and friends provide me with security; they bring me food and meals and take care of me. In addition to my family, friends and careers, these teams can promote my recovery; I am truly touched. Therefore, I told them that what I can do at present is to work hard, cooperate with them (physicians), and keep my body well. Therefore, I have no reason not to get well. In my family, my wife is over 70 years old, and she misses me every day... My wife and my kids, they formed a group to figure out how to take care of me... I have no reason not to get well". (P4)*

Another patient also reported a similar situation and the importance of family support:

*“My three children, they do not shirk their responsibilities to each other; they take care of me as soon as they are free”. (P5)*

Family support helps patients become confident and adhere to the rehabilitation and treatment program.

#### **Need for psychological support**

Many stroke patients experienced various levels of emotional distress after being ill, and they expressed their need for emotional support to varying degrees:

*“Sometimes you will have a bad mood all day for no reason, and you cannot control it at all. Sometimes I feel bad for no reason. I feel like I'm in a bad mood, but I cannot find any reason; somehow, I'm in a bad mood, and the family around me asked me what happened to you today? I do not know what happened to me. I was so upset... I felt the need to vent”. (P8)*

Psychological disturbances continue after stroke, and timely psychological intervention can significantly enhance the quality of life of patients.

#### **Peer support**

There is an old saying in China; that is, fellow patients support each other. For patients who have the same or similar experiences, support among patients is particularly important.

*“Actually, the patient with the surname Zhou was recovering well... He came back to the hospital today, and I didn’t recognize him yet. He’s just like a normal person. He got up and called to me, but I didn’t recognize him. He said you didn’t recognize me. I said I did not recognize him, but as soon as I walked out, he changed his clothes and was no longer wearing a patient gown. Then, I said that I truly didn’t recognize him. He told me who he was, and I remembered him right away. Learning from him, I feel that I will be recover well like him one day”. (P8)*

Effective support from peers provides hope for patients.

#### **Environmental support**

The daily activities of the stroke patients were limited due to physical limitations. During the rehabilitation process, a physical environment suitable for the activities and lives of rehabilitated patients should be created, and increasing space that patients can independently access can reduce their frustration and increase their sense of independence and control:

*“Can you communicate with the hospital and take measures to remove the walls and increase the number of scenes for everyone to exercise? There is no way to go out to exercise in autumn and winter. These settings are not conducive to the movement of patients”. (P11)*

This patient's statement highlights a broader need for environmental support that extends beyond the hospital walls. It powerfully illustrates how physical barriers‐whether in a clinical setting or the community‐can directly impede a patient's ability to practice autonomy, leading to increased frustration and a reinforced sense of dependence. Such experiences underscore the limitations of a fragmented, single‐intervention approach in rehabilitation and point to the necessity of developing a more holistic, multidimensional model of care.

### Regaining a partial sense of independence

Through gradual rehabilitation, the stroke patients gradually began to adjust their expectations and constantly adjusted their expectations thereafter, hoping to partly regain control over their lives and partial independence:

*“Being able to take care of myself and have a normal life. I don’t blame others for this. I was too sick, and I am semiparalysed...it’s definitely not the same as my past life. However, no matter how I treat it, it is impossible to use the same approach as in the past. I hope I can do something for self-care, including eating, bathing and self-caring”. (P4)*

With the support of their family members, many patients continued to make cognitive adjustments to help them better control their lives after stroke, and they began to gain some sense of control:

*“When you think of your family, thinking of them at home trying so hard to make money. I would feel sorry for them if I did not try to get better. It must be that the support of the family is very important; the family has to give you food but also to pay for expenses and to find someone to take care of you... When I think of these things, I adjust my emotions when I feel bad, and I encourage myself. When I do not want to exercise, I bite my teeth and stick to it, tell myself to exercise more and recover faster, tell myself that tomorrow will be better than today, and always think so... Give them hope, and give yourself hope, and you find that you can do more every day”. (P8)*

During the gradual recovery process, many patients saw changes in themselves and gradually gained a sense of control and independence:

*“I hope to be able to take care of myself, so as not to increase the burden on my family, as everyone has their own things (work and life difficulties) and no one can always take care of you. As long as you have the ability to take care of yourself, it is more convenient for others to help take care of you. If you cannot take care of yourself, you cannot do anything convenient, and you still need to work hard to recover. Of course, it will get better day by day; now, of course, I feel that there is hope because now I can walk. If you do not work hard on your own rehabilitation exercises and do not cooperate with the rehabilitation therapists or if the doctors are not good, you must strengthen your exercise. Rehabilitation is very hard, and not only relies on your own efforts but also on your own willpower... Only by working hard and getting better can I start living my life”. (P9)*

Many patients hope to regain control of their bodies and the ability to live independently and have control over their lives. To achieve this rehabilitation goal, the patients continuously made self‐adjustments at the cognitive, emotional, and behavioral levels and gradually gained some independence and a sense of control over their lives after stroke.

## Discussion

This study helped us understand how patients go from losing their sense of independence to gaining a partial sense of independence. This study revealed the realization path of multilevel holistic “environment, medical treatment, family, social psychology, peer relationships, etc.” rehabilitation services model.

First, the patients reported a loss of self and identity, as well as a loss of physical and cognitive ability after stroke, which all reflected the common experience of stroke patients with a loss of independence (Lou et al., 2017). Therefore, providing support regarding the physical, psychological, and social domains to help patients to regain personal, physical, cognitive, and interpersonal independence is the core focus of stroke patient care.

This study revealed that stroke patients have extensive limitations in their interpersonal relationships, social lives, and self‐care due to the physical dysfunction and disability caused by stroke, as well as a series of sequelae. The lack of independence reflected in the patients’ subjective statements not only indicated the sense of loss of control over their bodies but also reflected the sense of loss of control over their lives at a deeper level (Lou et al., 2017). Helping patients regain their sense of control and certainty about their bodies, lives, and interpersonal relationships is an important rehabilitation goal of patients after stroke.

Our study showed that the integration of rehabilitation services is very important, and our proposed multidimensional environmental‐medical‐family‐psychological‐relational rehabilitation approach is necessary for modern rehabilitation. Multisystem support helps patients gain a sense of mastery over their physical, psychological, and interpersonal relationships and the environment. Additionally, it enables patients to regain a sense of hope, which is considered a necessary condition for adaptation and recovery (Nott et al., 2021). Regarding physical rehabilitation, timely and effective rehabilitation treatment can help restore patients’ physical function to the maximum extent due to the receipt of timely and effective care. An increased sense of control over the body can help patients gain a better sense of hope. However, for stroke patients, recovery is incomplete in many cases, and patients are unable to regain a sense of control. In many cases, patients refuse to participate in rehabilitation, which undoubtedly hinders timely and effective recovery. Our study was consistent with previous studies. We found that timely and effective support provided by medical personnel can help give patients a sense of hope and improve their compliance and motivation in rehabilitation treatment, which can maximize the effect of rehabilitation. Moreover, effective family support also plays a critical role. An effective family support environment helps patients develop a sense of belonging and responsibility after stroke, gain a sense of purpose, and regain independence and confidence so that they seek help as soon as possible. Patients can also perform social activities and improve their quality of life (Mant et al., 2000). Notably, for stroke patients, creating a patient‐friendly environment is highly important. This is an important way for patients to gradually gain a sense of control over the environment. Patient‐friendly physical environments and settings enable patients to take control of their lives, reducing frustration and increasing independence. Previous studies have also confirmed that the interruption of social life after cerebrovascular disease onset increases the risk of developing psychological disturbances. Through psychological intervention and support, negative cognition in patients can be adjusted and eliminated, which can improve the efficacy of patient recovery (Yujuan et al., 2013).

Moreover, due to functional impairment after stroke, patients cannot return to their previous living habits. Previous studies have shown that homogeneous group life can help promote mutual learning of rehabilitation knowledge, goal setting, resource acquisition, problem solving and communication in patients and increase their emotional sense of belonging, control, and identity (Clark et al., 2020; Wallace et al., 2021). In future clinical practice, patients need to help establish a supportive peer group of rehabilitation patients, where mutual support and encouragement promote effective functional and psychosocial rehabilitation.

Previous studies have shown that a multidisciplinary collaborative optimization management model can improve the rehabilitation outcomes of patients with cerebrovascular diseases (Shenlin et al., 2021). Our study further revealed that comprehensive and multidimensional effective support for each dimension can help patients gain a better sense of control, and increased physical and social relationships and environmental support can promote partial independence.

### Limitations

This study has several limitations that warrant consideration. First, the findings may have limited transferability, as the research was conducted at a single university‐based hospital in Chongqing, China, and focused exclusively on the experiences of patients undergoing inpatient rehabilitation. The perspectives of patients after discharge may differ significantly.

Furthermore, as with all interview‐based research, participant accounts are subject to potential recall bias. Finally, while the sample of 13 participants was sufficient to achieve theoretical saturation for this grounded theory study, a larger and more diverse sample might have revealed additional nuances in the rehabilitation experience.

## Conclusion

It is pivotal to provide timely holistic rehabilitation services, as patients are more likely to receive timely and effective positive feedback. Effective positive feedback can help patients gradually gain a positive sense of control. Future rehabilitation services need to provide multidimensional services to enhance the facilitating factors in various environments and promote patients’ sense of control over their bodies, interpersonal relationships, and the environment. These visible changes can strengthen patients’ confidence and help them gain hope and independence in their lives.

## Consent for publication

Not applicable.

## Data Availability

The data will be made available upon request from the corresponding author.
